# De Novo Collapsing Glomerulopathy: An Unusual Cause of Early Graft Failure following Kidney Transplantation

**DOI:** 10.1155/2011/263970

**Published:** 2011-07-03

**Authors:** Kalathil K. Sureshkumar, Imran Dosani, Katherine M. Jasnosz, Swati Arora

**Affiliations:** ^1^Division of Nephrology and Hypertension, Department of Medicine, Allegheny General Hospital, Pittsburgh, PA 15212, USA; ^2^Department of Pathology, Allegheny General Hospital, Pittsburgh, PA 15212, USA

## Abstract

Collapsing glomerulopathy (CG) is a variant of focal segmental glomerulosclerosis (FSGS) characterized histologically by prominent glomerular capillary tuft collapse with hypertrophy and hyperplasia of podocytes and tubulointerstitial damage. Patients usually present with heavy proteinuria and rapidly progressive renal failure. We report a patient who developed de novo CG with severe clinical manifestations including worsening renal failure and nephrotic syndrome within six months of receiving deceased donor kidney transplant. Secondary work-up was negative, and despite therapy with high-dose steroids and plasmapheresis, allograft function rapidly deteriorated with the need for dialysis. Theories about the pathogenesis of this entity as well as treatment modalities are discussed.

## 1. Introduction

Collapsing glomerulopathy (CG) is a variant of focal segmental glomerulosclerosis (FSGS) characterized histologically by prominent glomerular capillary tuft collapse with hypertrophy and hyperplasia of podocytes and tubulointerstitial damage. Patients usually present with heavy proteinuria and rapidly progressive renal failure. Human immunodeficiency virus (HIV) is the most recognized cause of CG but non-HIV-associated CG is well described in both native kidneys and renal allografts [1, 2]. In renal allografts, CG can be recurrent or rarely de novo. Pathogenesis of CG is unknown. We report a renal transplant recipient who developed de novo CG with an aggressive course early after transplantation.

## 2. Clinical Presentation

A 56-year-old white female with hypertension and end-stage renal disease (ESRD) secondary to autosomal dominant polycystic kidney disease (ADPKD) and no prior sensitization underwent deceased donor kidney transplantation from a 52-year-old male who died from anoxic encephalopathy with terminal serum creatinine of 0.9 mg/dL. Donor's body mass index (BMI) was 49.6 kg/m^2^. Cold ischemia time was 19 hours, and there were 5 HLA mismatches. Patient received induction with single intravenous dose of alemtuzumab 30 mg intraoperatively and was initiated on tacrolimus (target through level 8–10 ng/mL)/mycophenolate mofetil (MMF, 500 mg twice daily)/early steroid withdrawal maintenance immunosuppression. Patient was started on trimethoprim-sulfamethoxazole and valganciclovir for Pneumocystis and cytomegalovirus (CMV) prophylaxes, respectively. There was prompt allograft function with a serum creatinine of 1.3 mg/dL (99.14 *μ*mol/L) at discharge 5 days after transplantation. The lowest serum creatinine achieved was 1.0 mg/dL (76.26 *μ*mol/L) at one month. Serum creatinine started to rise gradually starting at two months after transplantation. Two allograft biopsies were performed (days 75 and 90) showing no evidence of acute rejection or glomerular disease on light microscopy. A third biopsy of the allograft was performed 120 days after transplant due to further worsening of allograft function and generalized edema with a BUN of 45 mg/dL (16.1 mmol/L) and serum creatinine of 2.2 mg/dL (166.77 *μ*mol/L). Patient was also found to have 4.3 grams/day of proteinuria at this time. Her serum albumin decreased to 2.6 g/dL (26 g/L), and she developed severe hyperlipidemia with a fasting serum total cholesterol level of 468 mg/dL (12.1 mmol/L), features consistent with nephrotic syndrome. 

**Figure 1:**
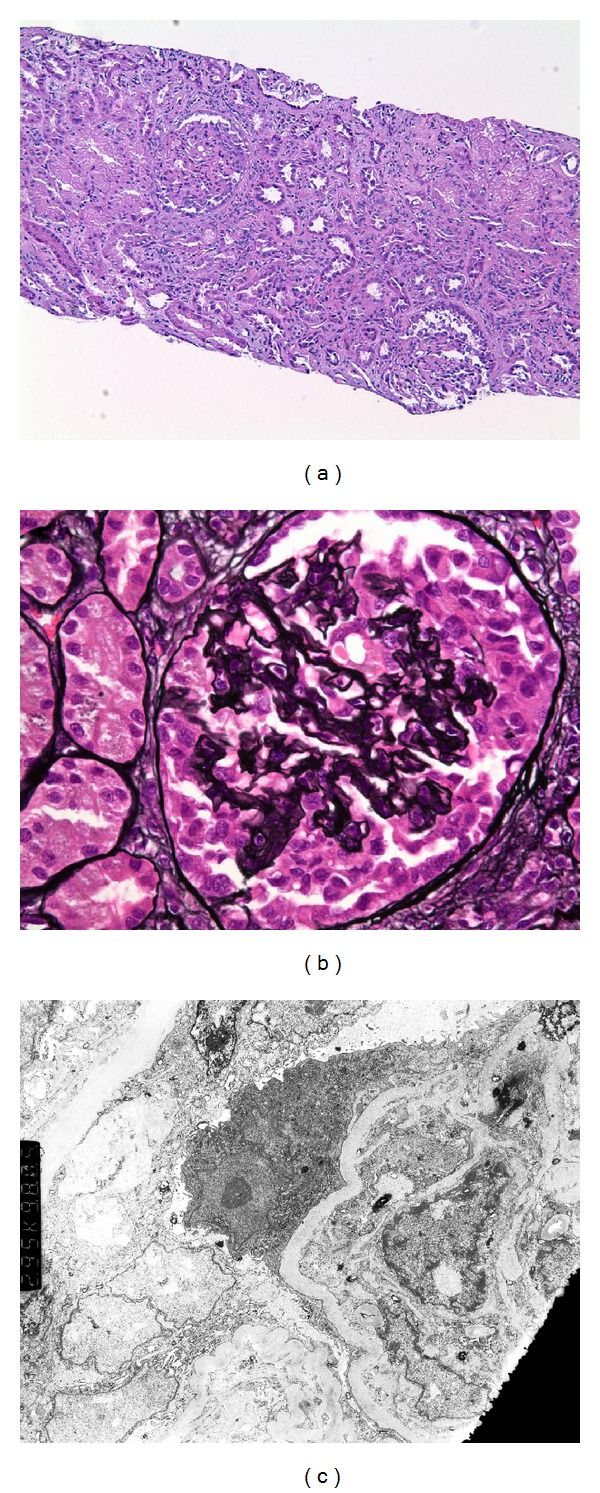
(a) Collapsed glomerular capillary loops with obliteration of vascular spaces are noted. Podocyte hyperplasia and synechiae formation along with tubular atrophy are evident (Hematoxylin-eosin 100x),(b) high-power view showing prominent collapse of glomerular tuft with obliteration of vascular spaces along with pseudocrescent formation (Jones-Silver stain 400x), and (c) electron microscopy showing collapse of the glomerular tuft with basement membrane wrinkling, podocyte enlargement, and effacement of foot processes. No tubuloreticular inclusions in endothelial cells are seen (2,950x).

Biopsy findings are shown in Figure 1.Figure 1(a) shows collapsed capillary loops in two glomeruli with complete obliteration of the vascular spaces. Hyperplasia of glomerular visceral epithelial cells (podocytes) with synechiae formation is evident. There is also evidence of tubuloepithelial damage and tubular atrophy with cystic dilatation. The glomerular changes were diagnostic of CG.  Figure 1(b) is higher-power view of a glomerulus showing prominent collapse of the glomerular tuft with obliteration of vascular space. Also evident is hypertrophy and hyperplasia of visceral epithelial cells (pseudocrescent), a hallmark of CG.  Figure 1(c) shows collapse of the tuft with basement membrane wrinkling, enlarged podocyte, and effacement of foot processes, changes consistent with CG. Endothelial tubuloreticular inclusions common in HIV nephropathy are not seen here. Immunofluorescence showed nonspecific entrapment of IgM and C3 in areas of collapse (figure not shown). Immunofluorescence was negative for C4d, and immunoperoxidase stain was negative for CMV and Polyoma virus.

Since the etiology of ESRD in our recipient was ADPKD, the biopsy findings were consistent with de novo rather than recurrent CG. Wedge biopsy of donor kidney at the time of organ procurement reported only 3 sclerotic glomeruli out of a total of 96 with no glomerular thrombi, mild interstitial fibrosis, and arteriolonephrosclerosis. Moreover, the mate kidney transplanted into another recipient has stable function without significant proteinuria making it less likely that donor-derived factors played a significant role in the pathogenesis of CG in our patient. 

We performed an extensive work-up to rule out secondary causes of CG. BMI of the patient was 30.7 kg/m^2^ at the time of transplantation and 31.1 kg/m^2^ at the time when CG was diagnosed, making obesity less likely the cause of CG. Patient has not been on bisphosphonates or interferon, medications known to be associated with development of CG. She gave no history of heroin use, and her tuberculin test was negative. Serologic tests including ANA, p-ANCA, c-ANCA, and hepatitis B surface antigen, hepatitis B core antibody, and hepatitis C antibody were negative. Serum immunofixation electrophoresis did not show any monoclonal bands. Similarly, HIV 1 and 2, Parvo virus B19 serologies, and serum CMV and BK Polyoma virus PCR were negative, infections known to be associated with development of CG. Although Parvo virus PCR is more sensitive than IgM and IgG levels, it was not done. In the absence of a secondary cause, we made a diagnosis of idiopathic de novo CG.

Based on the evidence of some beneficial effects of high-dose steroids in HIV-negative CG in native kidneys, we started the patient on prednisone 120 mg every other day once the diagnosis of CG was made. Maintenance immunosuppression with tacrolimus and MMF were continued. Therapy with lisinopril and simvastatin was started as well. Based on the known beneficial effects of plasmapheresis in recurrent FSGS following kidney transplantation in which the presence of permeability factor-like activity is implicated, we initiated the patient on plasmapheresis empirically on a three-times-per-week schedule. Despite these interventions, renal function continued to decline, and her proteinuria increased to 8.2 gram/day. The patient was initiated on maintenance hemodialysis a month later for uremic symptoms and volume overload. At that point, her BUN was 75 mg/dL (26.8 mmol/L), and serum creatinine was 4.6 mg/dL (350.80 *μ*mol/L). The patient received a total of 12 sessions of plasmapheresis by then. Her steroid dose was tapered slowly to a maintenance dose of 5 mg/day with discontinuation of both tacrolimus and MMF. She currently remains on maintenance hemodialysis.

## 3. Discussion

We present a case of de novo CG that presented as worsening allograft function with manifestations of nephrotic syndrome in a deceased donor kidney transplant resulting in allograft failure within six months of transplantation. Very early onset of the disease after transplantation and the fact that our patient is of White race and female gender are unique aspects of the case since there is a disproportionately higher incidence of CG in African Americans and in male gender. It is possible that initial two allograft biopsies had features of minimal change disease as precursor of CG that would be apparent only on electron microscopy which was not performed at that time.

Despite the generation of numerous hypotheses for the pathogenesis of CG, no specific common trigger for epithelial cell proliferation has emerged. The underlying pathogenic event appears to be a severe insult to the integrity and biology of glomerular visceral and parietal epithelial cells, resulting in cellular dedifferentiation and proliferation of these cells with loss of glomerular filtration barrier function [3–5]. At an ultrastructural level, disruption of mitochondrial functionality has been implicated as a common pathophysiological mechanism in CG [6]. Other postulated mechanisms include immune activation, hemodynamic disturbance at the arteriolar level, dysregulation of vascular endothelial growth factor expression, and acute ischemic processes from calcineurin inhibitor use [7–9]. 

A renal biopsy series spanning over an 18-year period reported five cases of de novo CG out of 892 allograft biopsies (0.6% of biopsies) with patients presenting 6–12 months after transplantation [10]. Four of these 5 patients were males. Our patient developed CG within 4 months of transplantation. A report from the Mayo Clinic analyzing their renal biopsy data base from 1994 to 2003 identified 10 cases of CG and 19 cases of noncollapsing FSGS among all renal allograft biopsies [11]. CG was more commonly observed in deceased donor kidneys, presented with more severe proteinuria and higher serum creatinine at diagnosis with more rapid graft loss without response to plasmapheresis.

No evidence-based therapy exists for either CG in native kidney or de novo CG in renal allografts. Survey of observational studies has reported only 9.6% complete remission and 15.2% partial remission with therapy for CG in native kidneys [4]. An open-label, nonrandomized pilot trial of intensive therapy with high-dose steroid and cyclosporine (intravenous for 2 weeks, then oral) along with prolonged course of plasmapheresis resulted in rapid, complete, and sustained remission in 9 of 10 patients presenting with recurrent FSGS after kidney transplantation [12]. It is, however, unclear whether plasmapheresis would yield significant benefit in patients with de novo CG following kidney transplantation.

In summary, we report the development of severe de novo CG in a renal allograft early after transplantation with an aggressive clinical course unresponsive to therapy resulting in allograft failure. De novo CG is rare in kidney transplants, its pathogenesis is unknown, and treatment is not defined. Prospective studies are needed to evaluate the effectiveness of plasmapheresis and other therapies in this disease.
